# Evaluation of the Eighth Edition of the American Joint Committee on Cancer TNM Staging System for Gastric Cancer: An Analysis of 7371 Patients in the SEER Database

**DOI:** 10.1155/2019/6294382

**Published:** 2019-04-14

**Authors:** Long-Long Cao, Jun Lu, Ping Li, Jian-Wei Xie, Jia-Bin Wang, Jian-Xian Lin, Qi-Yue Chen, Mi Lin, Ru-Hong Tu, Chao-Hui Zheng, Chang-Ming Huang

**Affiliations:** ^1^Department of Gastric Surgery, Fujian Medical University Union Hospital, Fuzhou, Fujian Province, China; ^2^Department of General Surgery, Fujian Medical University Union Hospital, Fuzhou, Fujian Province, China; ^3^Key Laboratory of Ministry of Education of Gastrointestinal Cancer, Fujian Medical University, Fuzhou, Fujian Province, China; ^4^Fujian Key Laboratory of Tumor Microbiology, Fujian Medical University, Fuzhou, Fujian Province, China

## Abstract

**Objective:**

To investigate the validity of the 8^th^ edition of the American Joint Committee on Cancer (AJCC) TNM staging system for gastric cancer.

**Methods:**

The clinicopathologic data of 7371 patients who were diagnosed with gastric cancer and had 16 or more involved lymph nodes (LNs) were retrieved from the Surveillance, Epidemiology, and End Results (SEER) database and retrospectively reviewed.

**Results:**

Stage migration occurred primarily during stage III between the 7^th^ and 8^th^ edition TNM staging systems. Stages IIIB and IIIC in the 7^th^ edition staging system were divided in the 8^th^ edition and had obvious differences in survival rates (both *P* < 0.001). The 8^th^ edition TNM stages IIIC and IV showed similar survival rates (*P* = 0.101). The prognosis of patients with T4aN3bM0 was not different from that of patients with TxNxM1 (*P* = 0.433), while the prognosis of patients with T4bN3bM0 was significantly poorer than that of patients with TxNxM1 (*P* = 0.008). A revised TNM system with both T4aN3bM0 and T4bN3bM0 incorporated into stage IV was proposed. Multivariable regression analysis showed that the revised TNM system, but not the 7^th^ and 8^th^ editions, was an independent factor for disease-specific survival (DSS) in the third step of the analysis. Further analyses revealed that the revised TNM system had superior discriminatory ability to the 8^th^ edition staging system, which was also an improvement over the 7^th^ edition staging system.

**Conclusion:**

The 8^th^ edition of the AJCC TNM staging system is superior to the 7^th^ edition for predicting the DSS rates of gastric cancer patients. However, for better prognostic stratification, it might be more suitable for T4aN3bM0/T4bN3bM0 to be incorporated into stage IV in the 8^th^ edition TNM staging system.

## 1. Introduction

Although its incidence is declining, gastric cancer remains one of the most common malignant tumors throughout the world and the second leading cause of cancer-related death worldwide [1, 2]. Accurate categorization of the tumor stage, including the invasive depth, lymph node (LN) metastasis and optimization of T and N categories, is crucial for prognostic assessment and decision-making of the stage-specific therapeutic strategy [3]. The American Joint Committee on Cancer (AJCC) TNM staging system for gastric cancer is the most important independent prognostic factor, and this system has been revised several times over the past three decades [4–7]. The 7^th^ edition of the AJCC staging system was published in 2010. Although many studies have demonstrated that the 7^th^ edition TNM staging system is better for prognostic prediction and has better reproducibility than the previous TNM staging systems, several limitations still exist [8, 9]. One such limitation is that although the N3 category was divided into N3a (7-15 involved regional LNs) and N3b (≥16 involved regional LNs), these divisions were not incorporated throughout the TNM staging system, which could reduce the efficiency of its prognostic prediction. Another limitation is that the prognosis of patients with disease classified into parts of stage IIIc according to the 7^th^ edition TNM system, including T4aN3bM0, T4bN3aM0, and T4bN3bM0, was similar to that of patients with stage IV, which reduced the prognostic stratification capacity between stage IIIC and stage IV. The 8^th^ edition of the AJCC TNM staging system was introduced in 2017 and reflected several changes from the 7^th^ edition, particularly regarding the combination of stage III [10–13]. Several studies [14–18] have reported that, compared with the 7^th^ edition, the 8^th^ edition provides better or comparable discrimination of overall survival differences among each TNM stage. However, little is known regarding the prognostic prediction ability of the 8^th^ edition staging system for disease-specific survival (DSS) in gastric cancer, especially for patients with 16 or more examined LNs.

In light of this evidence, we performed a retrospective study with 7371 gastric cancer patients selected from the Surveillance, Epidemiology, and End Results (SEER) Program database to evaluate the efficacy and validity of the 8^th^ edition of the AJCC TNM staging system for prognostic assessment and to provide guidelines for revising future editions of the AJCC staging system for gastric cancer staging.

## 2. Materials and Methods

### 2.1. Patients

A retrospective review of all gastric cancer patients from the SEER database between 1973 and 2013 was performed. A total of 99,253 patients from 18 SEER registries were initially screened. Cases were selected based on the primary site code (C16.0-C16.9, stomach) and associated histology codes (8021-8022, 8140, 8142-8145, 8210-8211, 8255, 8260-8263, 8323, 8480-8481, 8490, 8560, 8570, and 8574) [19, 20]. Patients with secondary tumors and negative histology were excluded. Patients were also excluded if they had not undergone lymphadenectomy (*n* = 24,834) or had fewer than 16 examined LNs (*n* = 62,065) or if incomplete information regarding T stage (*n* = 4918), N stage (*n* = 5), and M stage (*n* = 60) was available. Finally, a total of 7371 patients were enrolled in the study (Supplementary Figure 1).

### 2.2. Study Design

Patients' clinicopathological characteristics, such as age at diagnosis, gender, race, tumor site, tumor size, grade, T stage, N stage, and M stage, were collected. The pathological T stage, N stage, M stage, and final TNM stage were restaged to reflect the 7^th^ and 8^th^ editions of the AJCC staging system [10, 21]. Univariate survival analysis was used to assess the relationships between clinicopathological factors and DSS. To investigate the validity of the revised TNM system, a 3-step multivariate analysis was performed. In the 1^st^ step, all the factors that were significant in the univariate analysis as well as the 7^th^ edition TNM staging system were included; the 8^th^ edition and the revised staging system were excluded. In the 2^nd^ step, the 8^th^ edition TNM staging system was also included but not the revised staging system. Finally, in the 3^rd^ step, all three TNM systems were included.

### 2.3. Statistical Analysis

All enumeration and measurement data were analyzed using SPSS 17.0 for Windows. The univariate survival analysis was performed using the Kaplan-Meiermethod, and the significance of the differences between the groups was analyzed using the log-rank test. Stepwise multivariate survival analysis was performed using a Cox proportional hazards regression model to measure the independent contribution of each variable to survival. The concordance index (C-index), area under the curve (AUC) of the receiver operating characteristic (ROC) curve, and the Akaike information criterion (AIC) were used to measure the discriminatory ability of the models. For all analyses, only *P* < 0.05 was considered statistically significant.

## 3. Results

### 3.1. Patients and Demographics

For the present analysis, we enrolled 7371 gastric cancer patients from the SEER database who had 16 or more examined LNs and complete TNM staging and follow-up information. The majority of the patients were white (63.0%), 61.4% of the cohort was male, and the median age was 64 years. The total number of dissected LNs was 197,760, with an average of 26.8 ± 11.5 (mean ± standard deviation (SD)) dissected nodes per case. The mean number of metastatic nodes was 7.4 ± 9.5 overall. The median DSS for the entire cohort was 19 months (range, 0-119 months). The clinicopathological characteristics of all patients are listed in Supplementary Table 1.

### 3.2. Stage Migration

Table 1 reports changes in stage distribution between the 7^th^ and 8^th^ TNM classification systems for the overall gastric cancer group. Stages I, II, and IV showed almost no change. Only 3.4% of the cases classified by the 7^th^ edition as stage IIIA shifted to be classified by the 8^th^ edition as stage IIIB, whereas 44.2% of the stage IIIC cases shifted to stage IIIB according to the 8^th^ edition TNM system. Cases that were classified as stage IIIB using the 7^th^ edition system shifted either to the more advanced stage IIIC (23.8%) or to stage IIIA (24.1%) with the 8^th^ edition TNM system. As shown, there were significant differences in survival for the 7^th^ edition stage IIIB and IIIC (both *P* < 0.001) patients but not for the stage IIB (*P* = 0.221) and IIIA patients (*P* = 0.458) stratified according to the 8^th^ edition TNM system. However, when stratified according to the 7^th^ edition TNM system, significant differences in survival could not be observed for the 8^th^ edition stage IIIA (*P* = 0.091) patients, but the 8^th^ edition stage IIIB (*P* = 0.029) and IIIC (*P* = 0.006) patients showed significant differences.

To investigate whether the number of examined LNs affected the stage migration from the 7^th^ edition system to the 8^th^ edition, patients were divided into two groups according to the total number of examined LNs [12]: 5320 patients with 16-29 examined LNs and 2051 patients with ≥30 examined LNs. As shown in Supplementary Table 2 and Supplementary Figure 2, for patients with 16-29 examined LNs, patients with disease classified by the 7^th^ edition as stage IIIB and IIIC disease (both *P* < 0.001) had significant differences in survival when stratified according to the 8^th^ edition system, which was consistent with the results obtained from the overall cohort. Interestingly, for patients with ≥30 examined LNs, there were significant differences in survival for those classified by the 7^th^ edition with stages IIB, IIIB, and IIIC disease (all *P* < 0.001) but not for those classified by the 8^th^ edition system with stage IIIA disease (*P* = 0.826). However, when stratified according to the 7^th^ edition system, significant differences in survival could not be observed for all the subgroup of the 8^th^ edition stage III patients (all *P* > 0.05) (Supplementary Table 3 and Supplementary Figure 2). These results suggested that the 8^th^ edition system could provide better accuracy than the 7^th^ edition for stage III stratification, especially for patients with ≥30 examined LNs.

### 3.3. Survival Analysis for the 7^th^ and 8^th^ Edition TNM Systems

**Figure 1:**
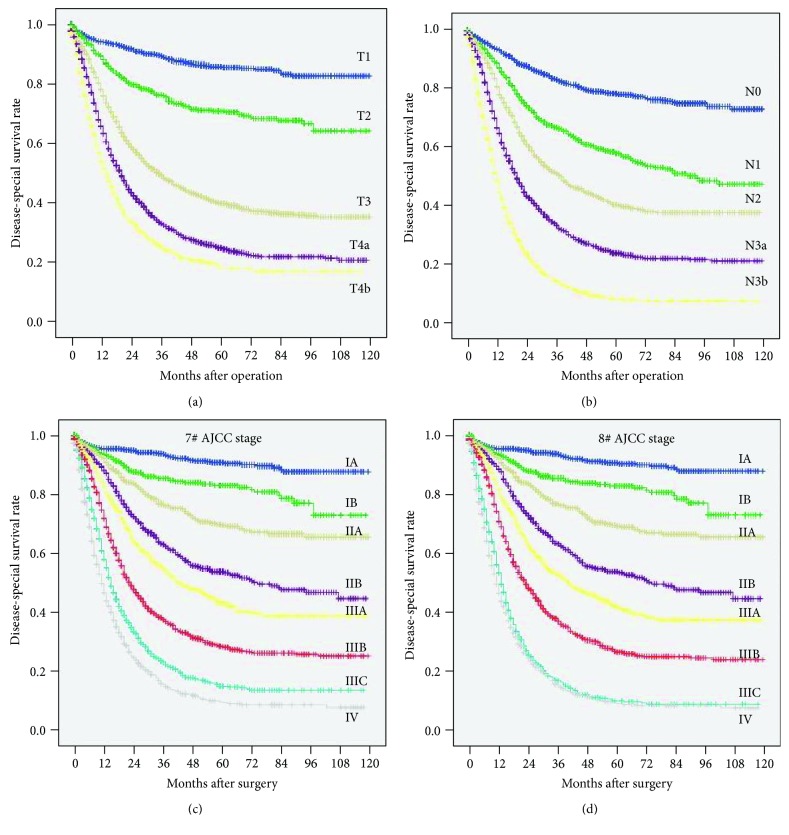
Comparison of survival curves. (a) According to the 8^th^ edition T category. (b) According to the 8^th^ edition N category. (c) According to the 7^th^ edition AJCC TNM stage. (d) According to the 8^th^ edition AJCC TNM stage.

**Figure 2:**
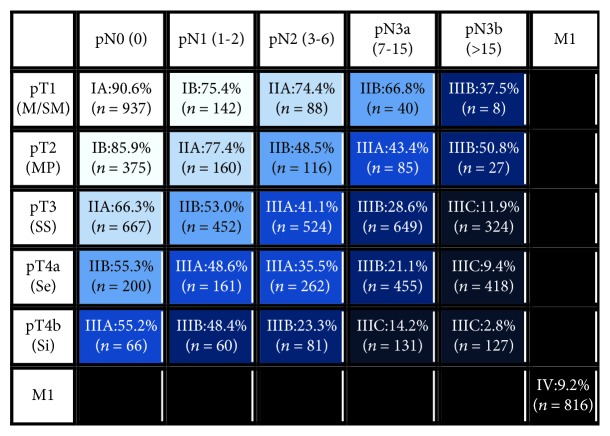
Five-year survival rates according to the 8^th^ edition T and N categories.

Comparisons of survival curves among patients with different T and N categories according to the 7^th^ and 8^th^ edition TNM systems are presented in Figures 1(a) and 1(b). Significant differences in prognosis were observed, including those between patients with the N3a and N3b categories (*P* < 0.001). Additionally, significantly different survival rates were observed among most of the groups classified by the two different editions of the TNM system (all *P* < 0.001), except between patients with stages IIIC and IV disease as classified by the 8^th^ edition (Figures 1(c) and 1(d)). The 5-year survival rates according to the T and N categories are shown in Figure 2. For the patients with disease in each T category, survival was significantly different among patients with disease in different N categories (all *P* < 0.001). Similarly, for the patients with disease in each N category, survival was significantly different among patients with disease in different T categories (all *P* < 0.001). Moreover, significant differences in prognosis between the N3a and N3b categories were observed for patients with disease in the T3 (*P* < 0.001), T4a (*P* < 0.001), and T4b (*P* < 0.001) categories but not for those with disease in the T1 (*P* = 0.332) and T2 (*P* = 0.610) categories.

### 3.4. Revised TNM System

**Figure 3:**
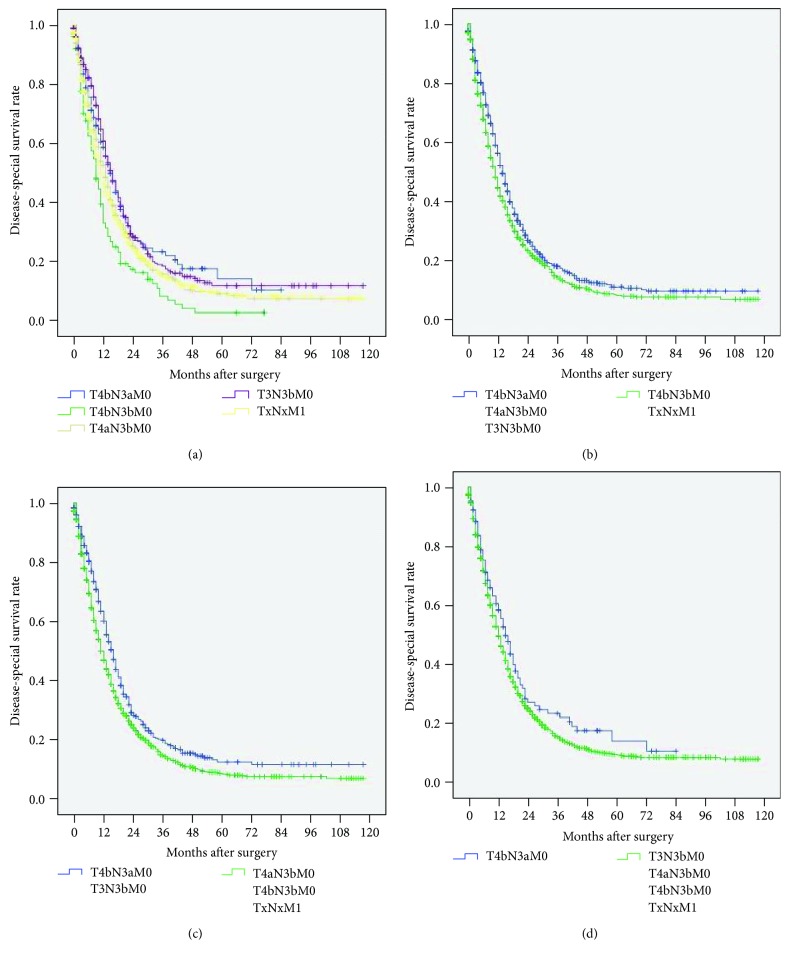
Comparison of survival curves based on the 8^th^ edition staging system. (a) Among T4bN3aM0/T4bN3bM0/T4aN3bM0/T3N3bM0/TxNxM1. (b) Between T4bN3aM0/T4aN3bM0/T3N3bM0 and T4bN3bM0/TxNxM1 (HR: 1.047, 95% CI: 1.020-1.076, *P* = 0.001). (c) Between T4bN3aM0/T3N3bM0 and T4aN3bM0/T4bN3bM0/TxNxM1 (HR: 1.062, 95% CI: 1.029-1.097, *P* < 0.001). (d) Between T4bN3aM0 and T4aN3bM0/T4bN3bM0/T3N3bM0/TxNxM1 (HR: 1.048, 95% CI: 0.993-1.106, *P* = 0.089).

In the 8^th^ edition TNM system, patients with stages IIIC and IV disease showed similar survival rates (*P* = 0.101, Figure 1(d)). Further analyses showed that the prognosis of patients with T4bN3aM0/T3N3bM0 disease was significantly better than that of patients with TxNxM1 disease (*P* = 0.044 and *P* = 0.002, Figure 3(a)), and the prognosis of patients with T4bN3bM0 disease was significantly worse than that of patients with TxNxM1 disease (*P* = 0.008, Figure 3(a)). Additionally, the prognosis of patients with T4aN3bM0 stage disease was not significantly different from that of patients with TxNxM1 stage disease (*P* = 0.433, Figure 3(a)). We hypothesized that T4bN3bM0, T4aN3bM0, and T3N3bM0 were incorporated step by step into stage IV so that the prognosis of patients with T4bN3bM0/TxNxM1 stage disease was significantly different from that of patients with T4aN3bM0/T4bN3aM0/T3N3bM0 stage disease (hazard ratio (HR): 1.047, 95% confidence interval (CI): 1.020-1.076, *P* = 0.001, Figure 3(b)); the prognosis of patients with T4bN3bM0/T4aN3bM0/TxNxM1 stage disease was significantly different from that of patients with T4bN3aM0/T3N3bM0 stage disease (HR: 1.062, 95% CI: 1.029-1.097, *P* < 0.001, Figure 3(c)), and the prognosis of patients with T4bN3bM0/T4aN3bM0/T3N3bM0/TxNxM1 stage disease was significantly different from that of patients with T4bN3aM0 stage disease (HR: 1.048, 95% CI: 0.993-1.106, *P* = 0.089, Figure 3(d)). Based on these results, we proposed a revised TNM system in which both T4aN3bM0 and T4bN3bM0 were incorporated into stage IV.

### 3.5. Univariate Analysis and 3-Step Multivariate Analysis

In the univariate analysis, age, race, tumor site, tumor size, grade, the 7^th^ edition TNM system, the 8^th^ edition TNM system, and the revised TNM system were significantly correlated with survival (all *P* < 0.05). In the 1^st^ step of multivariate analysis, age, race, tumor size, grade, and the 7^th^ edition TNM system were confirmed to be independent prognostic factors (all *P* < 0.05). When the 8^th^ edition TNM system was included in the 2^nd^ step of multivariate analysis, it was also confirmed to be an independent prognostic factor (*P* < 0.05). However, when all three TNM systems were analyzed in the 3^rd^ step, only the revised TNM system but not the 7^th^ or 8^th^ TNM staging systems was an independent prognostic factor (*P* < 0.05) (Table 2).

### 3.6. The Revised TNM System Has Better Prognostic Stratification Than the Other Two Systems

The performances of all three TNM stage systems were assessed using the C-index, AUC, and AIC (Table 3 and Supplementary Figure 3). The 8^th^ edition TNM system had a higher C-index (7^th^ edition vs. 8^th^ edition: 0.725 vs. 0.734), a higher AUC (0.770 vs. 0.773), and a smaller AIC value (56,463.140 vs. 56,396.524) than the 7^th^ edition TNM system. A similar result was observed for stage III of the 8^th^ edition compared with stage III of the 7^th^ edition. However, the revised system had a higher C-index (8^th^ edition vs. revised system: 0.734 vs. 0.741), a higher AUC (0.773 vs. 0.774), and a smaller AIC value (56,396.524 vs. 56,355.250) than the 8^th^ edition TNM system. Taken together, these results revealed that the revised TNM system had superior discriminatory ability to the 8^th^ edition system, which was also an improvement over the 7^th^ edition system.

## 4. Discussion

The TNM classification of cancer is the most important independent prognostic factor and is considered to play a fundamental role in treatment. For gastric cancer, several editions of the TNM system have been published in the past 30 years. Major changes in these editions have mainly focused on both the T staging system and the N staging system [7, 21]. The 7^th^ edition of the AJCC TNM classification system released in 2010 has proven to be an excellent classification system and has been extensively used for gastric cancer staging worldwide. However, several problems, including N3a and N3b subcategories that do not require individual determinants in the final TNM stage, have been associated with its use [8, 22]. Consequently, the 8^th^ edition of the AJCC TNM classification system for gastric cancer was published in 2017 and included major revisions in the N classifications and stage III. However, the validity of these revisions remains unknown.

In the present study, significant differences in survival were observed among patients with disease in different T categories according to the 8^th^ edition staging system. Moreover, for patients with disease in each N category, there were significant differences in prognosis among those with disease in different T categories. These results indicate that the T staging system in the 8^th^ edition has excellent prognostic stratification. Although the same subcategories of the N staging system are used in the 7^th^ and 8^th^ editions, the N3a and N3b categories are individual determinants of the final TNM stage in the 8^th^ edition but not in the 7^th^ edition. In the present study, for the 8^th^ edition N staging system, significant differences in survival were observed between patients with disease in the N3a and N3b categories and among those with disease in different N categories. Additionally, for patients with disease in each T category, there were significant differences in prognosis among those with disease in different N categories, including the N3a and N3b categories. Our data are consistent with those of previous studies, which indicated that the prognosis of patients with more than 15 metastatic LNs is significantly worse than that of patients with 7-15 metastatic LNs [9, 23, 24]. These results suggest that it is reasonable to subclassify the N3 category into N3a and N3b subcategories and, more importantly, to include different determinants in the final TNM system.

In addition, stage migration mainly occurred in stage III between the 7^th^ and 8^th^ TNM systems. There were significant differences in survival among patients with disease classified by the 7^th^ edition as stages IIIB and IIIC disease when they were stratified using the 8^th^ edition TNM system. This result was similar to previous data showing that the overall survival rates were significantly different in patients with stages IIIB and IIIC disease as stratified by the 8^th^ system whose disease was classified by the 7^th^ edition system as stage IIIB disease [11]. However, no significant differences in prognosis were observed among patients with disease classified by the 8^th^ edition as stage IIIA disease when they were stratified using the 7^th^ edition TNM system. Moreover, the prognostic stratification for stage III disease according to the 8^th^ edition system was superior to that of the 7^th^ edition in terms of the HR (data not shown). These results further indicate that the 8^th^ edition system can provide more reasonable classification with more power than the 7^th^ edition system to subclassify patients with more homogenous prognoses.

However, drawbacks persist in the 8^th^ edition of the TNM system. As the present study shows, there were no significant differences between the 8^th^ edition stages IIIC and IV in terms of DSS rates. Moreover, similar prognoses were observed between patients with T4bN3aM0/T4bN3bM0/T4aN3bM0 and TxNxM1 disease when the prognoses of patients with disease in stage IIIC and stage IV subcategories were analyzed. Through the step-by-step incorporation of T4bN3bM0, T4aN3bM0, and T4bN3aM0 into stage IV, we further confirmed that the best prognostic stratification of stages IIIC and IV occurred when T4bN3bM0 and T4aN3bM0 were incorporated into stage IV. According to these results, we propose a revision of the 8^th^ edition TNM staging system in which both T4bN3bM0 and T4aN3bM0 are incorporated into stage IV because of their similar prognoses.

To investigate the validity of the revised TNM system, a 3-step multivariate analysis was performed. In the 2^nd^ step of the analysis, both the 7^th^ and the 8^th^ edition systems were confirmed to be independent predictors of patient survival. Additionally, compared with the 7^th^ TNM system and its stage III, both the 8^th^ TNM system and its stage III had better or at least comparable discriminatory ability, which was consistent with the findings of previous studies [12, 13]. These results indicate that the 8^th^ TNM system is superior to the 7^th^ system for predicting the 5-year DSS rates of gastric cancer patients due to the optimum prognostic stratification of stage III disease. However, when the revised system was also included in the 3^rd^ step of the analysis, it became an independent predictor of survival, whereas both the 7^th^ and 8^th^ edition staging systems were no longer independent predictors. Most importantly, the proposed system had a higher C-index, a higher AUC, and a smaller AIC value than the 7th and 8th edition systems, which suggested that the revised system had superior discriminatory ability to the 7^th^ and 8^th^ edition systems. Although there were no extreme differences between the revised system and the 8^th^ edition system, the revised system indeed had superior prognostic stratification for stage IIIC and stage IV disease. Therefore, our results demonstrated that the revised system is superior to the 7^th^ and 8^th^ edition systems for prognostic assessment.

The main limitation of the current study is its retrospective analysis setting. The impact of various treatment-related outcomes could not be evaluated fully in this study. Despite this limitation, our data provide important insight into the application of the 8^th^ edition system and the revised TNM classification system for gastric cancer patients. Our study could be the basis for a subsequent prospective clinical study.

In conclusion, the present study demonstrates that the 8^th^ TNM system is superior to the 7^th^ system for predicting the 5-year DSS rates of gastric cancer patients due to the optimal prognostic stratification of stage III disease. However, for better prognostic stratification, we propose a revised TNM system in which T4aN3bM0 and T4bN3bM0 are incorporated into stage IV. Since the revised system was superior to the 8^th^ edition system in terms of its discriminatory ability, we recommended that this system be considered for clinical application. Further studies should be carried out to confirm our results.

## Figures and Tables

**Table 1:** Comparison of the 5-year survival rates based on the 7^th^ edition of the TNM system and the 8^th^ edition of the TNM system.

7th edition	8th edition (5-YSR)								*P* value
	IA	IB	IIA	IIB	IIIA	IIIB	IIIC	IV	
IA	937								N/A
IB		517							N/A
IIA			915						N/A
IIB				808 (53.8%)		8 (37.5%)			0.221
IIIA					770 (43.0%)	27 (50.8%)			0.458
IIIB					328 (39.5%)	709 (30.3%)	324 (11.9%)		<0.001
IIIC						536 (21.4%)	676 (9.2%)		<0.001
IV								816	N/A
*P* value	N/A	N/A	N/A	N/A	0.091	0.029	0.006	N/A	

**Table 2:** Univariate analysis and 3-step multivariate analysis of prognostic factors for gastric cancer patients.

	Univariate analysis			Multivariate analysis 1st			Multivariate analysis 2nd			Multivariate analysis 3rd		
	HR	95% CI	*P* value	HR	95% CI	*P* value	HR	95% CI	*P* value	HR	95% CI	*P* value
Age	1.234	1.151-1.322	<0.001	1.472	1.372-1.579	<0.001	1.473	1.374-1.580	<0.001	1.453	1.355-1.559	<0.001
Gender	1.038	0.967-1.115	0.297									
Race	0.866	0.831-0.903	<0.001	0.880	0.844-0.918	<0.001	0.880	0.844-0.918	<0.001	0.876	0.840-0.913	<0.001
Tumor site	1.018	0.999-1.037	0.059									
Tumor size	1.373	1.310-1.440	<0.001	1.139	1.080-1.200	<0.001	1.135	1.077-1.197	<0.001	1.120	1.062-1.182	<0.001
Grade	1.220	1.167-1.275	<0.001	1.113	1.056-1.174	<0.001	1.110	1.052-1.170	<0.001	1.099	1.042-1.159	0.001
TNM stage (AJCC 7th)	1.511	1.482-1.541	<0.001	1.504	1.475-1.534	<0.001	1.081	1.002-1.166	0.044			
TNM stage (AJCC 8th)	1.530	1.500-1.560	<0.001				1.413	1.310-1.524	<0.001			
Revised TNM system	1.501	1.482-1.511	<0.001							1.333	1.249-1.410	<0.001

**Table 3:** Comparison of the performance of various editions of the TNM staging system.

Model	Concordance index		ROC curve		AIC
	C-index	95% CI	AUC	95% CI	
TNM stage (AJCC 7th)	0.725	0.717-0.740	0.770	0.759-0.781	56,463.140
TNM stage (AJCC 8th)	0.734	0.720-0.741	0.773	0.762-0.783	56,396.524
Stage III (AJCC 7th)	0.594	0.581-0.607	0.607	0.587-0.626	31,552.480
Stage III (AJCC 8th)	0.608	0.599-0.624	0.621	0.602-0.640	31,515.240
Revised TNM system	0.741	0.730-0.748	0.774	0.763-0.784	56,355.250

ROC curve: receiver operating characteristic curve; AUC: area under curve; 95% CI: 95% confidence interval; AIC: Akaike information criterion.

## Data Availability

The data used to support the findings of this study are available from the corresponding author upon request.

## Supplementary Materials

Supplementary Materials Supplementary Figure 1: flow diagram of patient inclusion and exclusion. Supplementary Figure 2: comparison of survival curves according to the total examined lymph nodes (ELN). (A) The 7th edition AJCC stage for patients with 16-29 ELN. (B) The 8th edition AJCC stage for patients with 16-29 ELN. (C) The 7th edition AJCC stage for patients with ≥30 ELN. (D) The 8th edition AJCC stage for patients with ≥30 ELN. Supplementary Figure 3: predictive values of 7th and 8th edition AJCC stage and the revised system by receiver operating characteristic (ROC) curve. The area under curve (AUC) of the 7th and 8th edition AJCC stage and the revised system is 0.770 (95% IC: 0.759-0.781), 0.773 (0.762-0.783), and 0.774 (0.763-0.784), respectively. Supplementary Table 1: demographics and clinical characteristics of study patients. Supplementary Table 2: comparison of 5-year survival rate based on the 7th edition system according to the 8th edition system for patients with 16-29 examined lymph nodes. Supplementary Table 3: comparison of 5-year survival rate based on the 7th edition system according to the 8th edition system for patients with ≥30 examined lymph nodes.
