# Supplementary material for: Evaluation of the Eighth Edition of the American Joint Committee on Cancer TNM Staging System for Gastric Cancer: An Analysis of 7371 Patients in the SEER Database
Source: Gastroenterol Res Pract. 2019 Apr 14;2019:6294382. doi: 10.1155/2019/6294382 (PMC6487090; doi:10.1155/2019/6294382)
Supplement: Supplementary Materials — Supplementary Figure 1: flow diagram of patient inclusion and exclusion. Supplementary Figure 2: comparison of survival curves according to the total examined lymph nodes (ELN). (A) The 7th edition AJCC stage for patients with 16-29 ELN. (B) The 8th edition AJCC stage for patients with 16-29 ELN. (C) The 7th edition AJCC stage for patients with ≥30 ELN. (D) The 8th edition AJCC stage for patients with ≥30 ELN. Supplementary Figure 3: predictive values of 7th and 8th edition AJCC stage and the revised system by receiver operating characteristic (ROC) curve. The area under curve (AUC) of the 7th and 8th edition AJCC stage and the revised system is 0.770 (95% IC: 0.759-0.781), 0.773 (0.762-0.783), and 0.774 (0.763-0.784), respectively. Supplementary Table 1: demographics and clinical characteristics of study patients. Supplementary Table 2: comparison of 5-year survival rate based on the 7th edition system according to the 8th edition system for patients with 16-29 examined lymph nodes. Supplementary Table 3: comparison of 5-year survival rate based on the 7th edition system according to the 8th edition system for patients with ≥30 examined lymph nodes. [file 6294382.f1.docx]

| Supplementary Table 1. Demographics and Clinical Characteristics of Study Patients. | | |
| --- | --- | --- |
| Characteristic | No. of Patients | % |
| Age (Y) |  |  |
| Mean | 63.9 |  |
| SD | 13.2 |  |
| Gender |  |  |
| Male | 4523 | 61.4 |
| Female | 2848 | 38.6 |
| Race |  |  |
| White | 4643 | 63.0 |
| Black | 895 | 12.2 |
| Other | 1794 | 24.3 |
| Unknown | 39 | 0.5 |
| Tumor site |  |  |
| Upper | 2114 | 28.7 |
| Middle | 727 | 9.9 |
| Lower | 2016 | 27.4 |
| Mixed | 676 | 9.2 |
| Lesser curvature | 911 | 12.3 |
| Greater curvature | 363 | 4.9 |
| Unknown | 564 | 7.6 |
| Tumor size |  |  |
| ≤5.4cm | 3660 | 49.7 |
| ＞5.4cm | 2846 | 38.6 |
| Unknown | 865 | 11.7 |
| Grade |  |  |
| Well | 245 | 3.3 |
| Moderately | 1694 | 23.0 |
| Poorly | 4950 | 67.2 |
| Undifferentiated | 201 | 2.7 |
| Unknown | 281 | 3.8 |
| T status |  |  |
| T1 | 1235 | 16.8 |
| T2 | 783 | 10.6 |
| T3 | 2879 | 39.1 |
| T4a | 1822 | 24.7 |
| T4b | 652 | 8.8 |
| N status |  |  |
| N0 | 2288 | 31.0 |
| N1 | 1022 | 13.9 |
| N2 | 1176 | 16.0 |
| N3a | 1621 | 22.0 |
| N3b | 1264 | 17.1 |
| M status |  |  |
| M0 | 6555 | 88.9 |
| M1 | 816 | 11.1 |
| TNM stage (AJCC 8th) |  |  |
| IA | 937 | 12.7 |
| IB | 517 | 7.0 |
| IIA | 915 | 12.4 |
| IIB | 808 | 11.0 |
| IIIA | 1098 | 14.9 |
| IIIB | 1280 | 17.4 |
| IIIC | 1000 | 13.6 |
| IV | 816 | 11.0 |

| Supplementary Table 2. Comparison of 5-year survival rate based on the 7th edition system according to the 8th edition system for patients with 16-29 examined lymph nodes. | | | | | | | | | |
| --- | --- | --- | --- | --- | --- | --- | --- | --- | --- |
|  | The 8th edition (5-YSR) | | | | | | | |  |
|  | IA | IB | IIA | IIB | IIIA | IIIB | IIIC | IV | P Value |
| The 7th edition |  |  |  |  |  |  |  |  |  |
| IA | 692 |  |  |  |  |  |  |  | N/A |
| IB |  | 383 |  |  |  |  |  |  | N/A |
| IIA |  |  | 700 |  |  |  |  |  | N/A |
| IIB |  |  |  | 603(52.5%) |  | 3(100%) |  |  | 0.314 |
| IIIA |  |  |  |  | 575(40.6%) | 15(43.1%) |  |  | 0.350 |
| IIIB |  |  |  |  | 247(37.2%) | 524(27.2%) | 140(10.4%) |  | <0.001 |
| IIIC |  |  |  |  |  | 415(18.7%) | 412(6.0%) |  | <0.001 |
| IV |  |  |  |  |  |  |  | 611 | N/A |
| *P* Value | N/A | N/A | N/A | N/A | 0.105 | 0.015 | 0.002 | N/A |  |
|  |  |  |  |  |  |  |  |  |  |

| Supplementary Table 3. Comparison of 5-year survival rate based on the 7th edition system according to the 8th edition system for patients with ≥30 examined lymph nodes. | | | | | | | | | |
| --- | --- | --- | --- | --- | --- | --- | --- | --- | --- |
|  | The 8th edition (5-YSR) | | | | | | | |  |
|  | IA | IB | IIA | IIB | IIIA | IIIB | IIIC | IV | P Value |
| The 7th edition |  |  |  |  |  |  |  |  |  |
| IA | 245 |  |  |  |  |  |  |  | N/A |
| IB |  | 134 |  |  |  |  |  |  | N/A |
| IIA |  |  | 215 |  |  |  |  |  | N/A |
| IIB |  |  |  | 205(57.9%) |  | 5(6.0%) |  |  | <0.001 |
| IIIA |  |  |  |  | 195(50.7%) | 12(65.5%) |  |  | 0.826 |
| IIIB |  |  |  |  | 81(49.2%) | 185(39.6%) | 184(13.0%) |  | <0.001 |
| IIIC |  |  |  |  |  | 121(31.1%) | 264(14.1%) |  | <0.001 |
| IV |  |  |  |  |  |  |  | 205 | N/A |
| *P* Value | N/A | N/A | N/A | N/A | 0.615 | 0.084 | 0.971 | N/A |  |
|  |  |  |  |  |  |  |  |  |  |
|  |  |  |  |  |  |  |  |  |  |


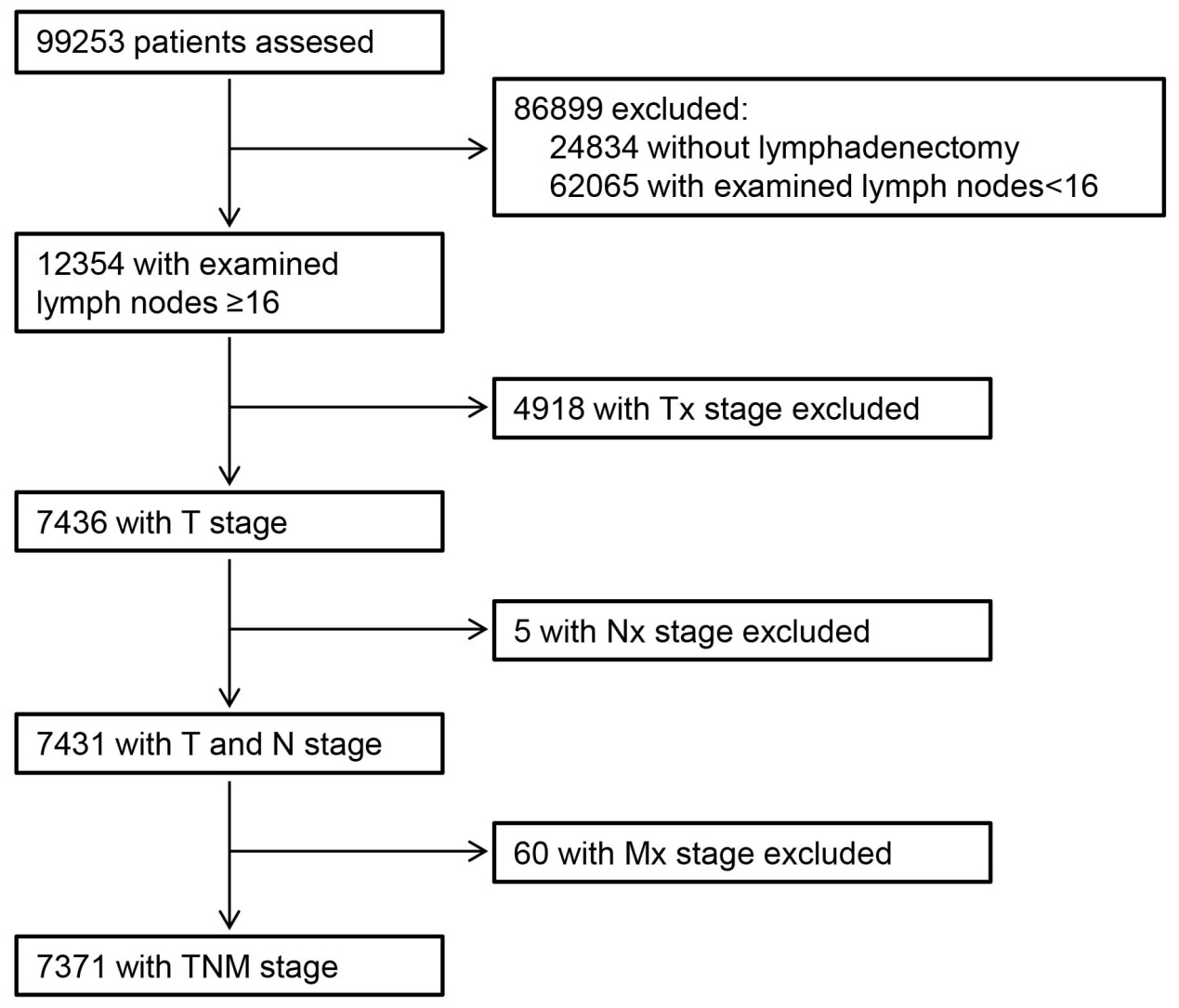


**Supplementary Figure 1.** Flow diagram of patient inclusion and exclusion**.**


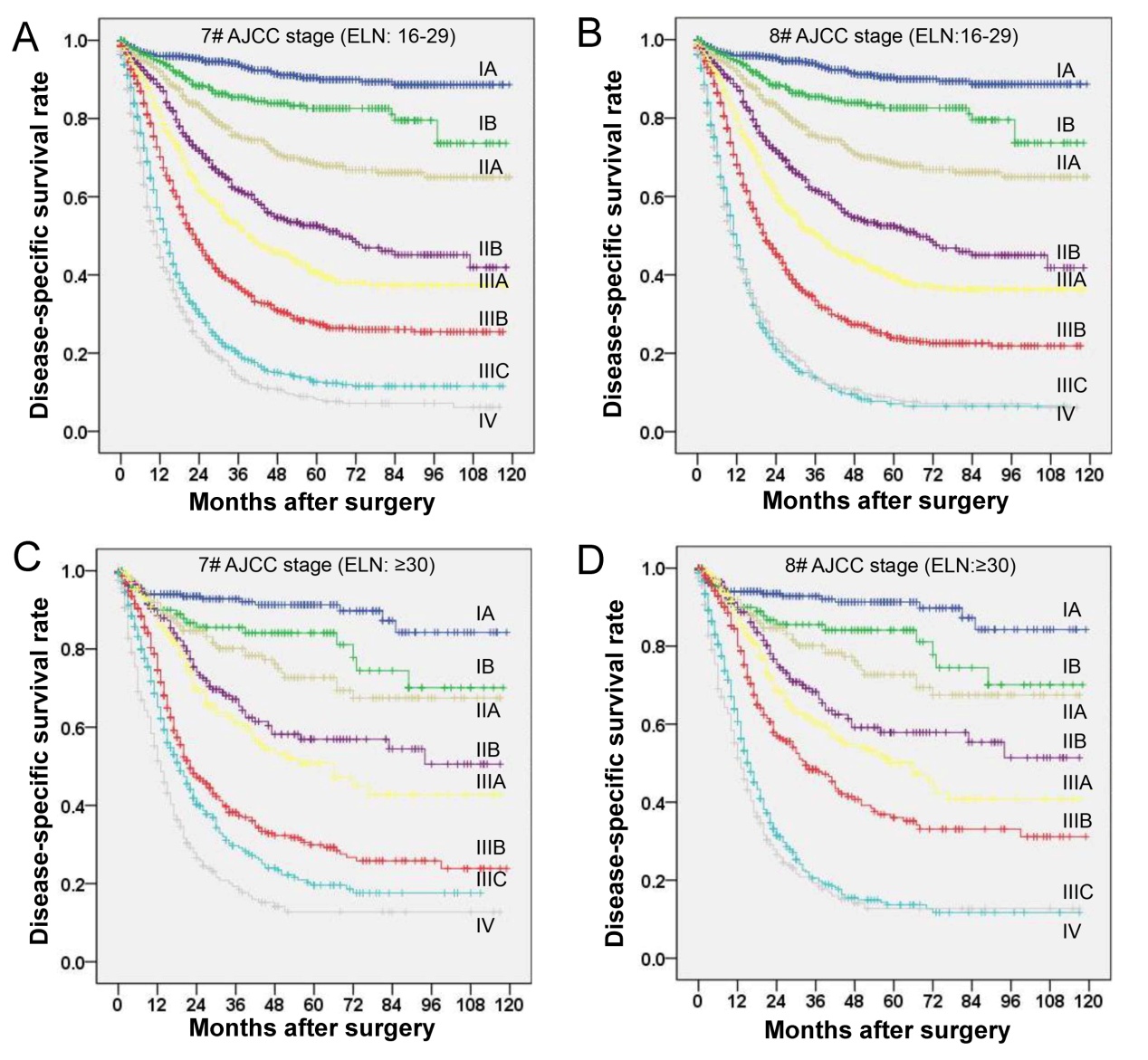


**Supplementary Figure 2.** Comparison of survival curves according to the total examined lymph nodes (ELN). **A** The 7^th^ edition AJCC stage for patients with 16-29 ELN. **B** The 8^th^ edition AJCC stage for patients with 16-29 ELN. **C** The 7^th^ edition AJCC stage for patients with ≥30 ELN. **D** The 8^th^ edition AJCC stage for patients with ≥30 ELN.


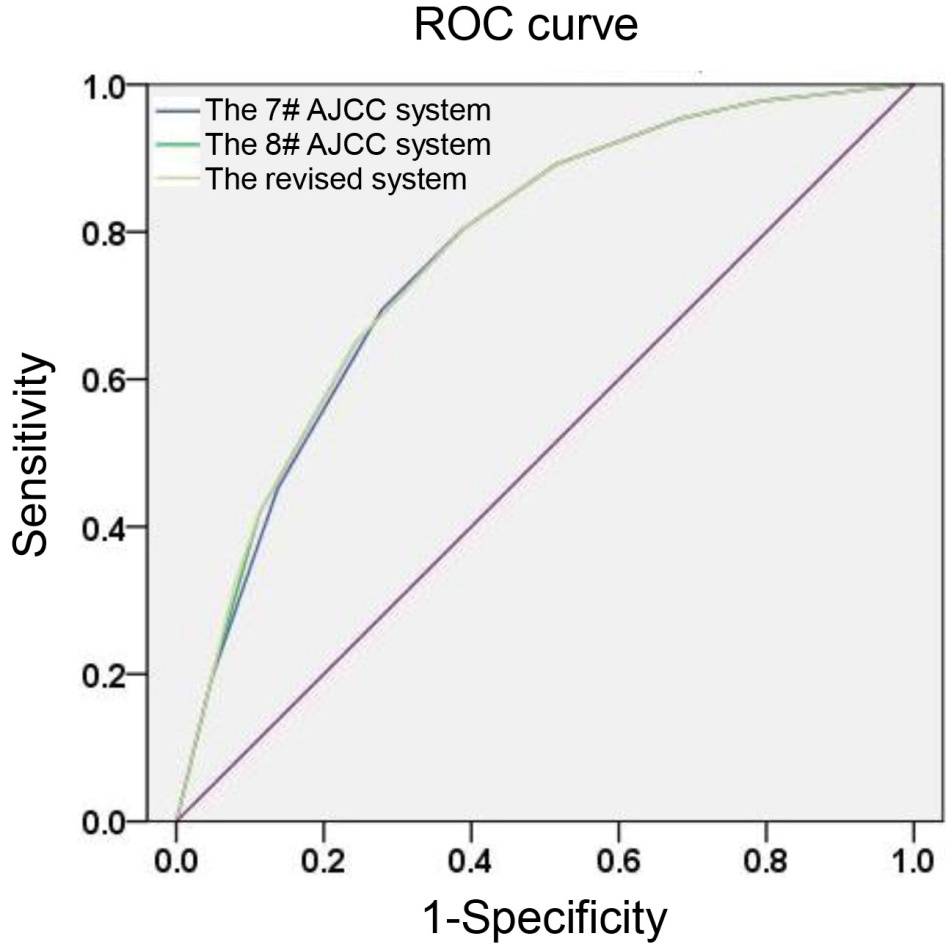


**Supplementary Figure 3.** Predictive values of 7^th^, 8^th^ edition AJCC stage and the revised system by receiver operating characteristic (ROC) curve**.** The area under curve (AUC) of the 7^th^, 8^th^ edition AJCC stage and the revised system is 0.770 (95% IC: 0.759-0.781), 0.773 (0.762-0.783), and 0.774 (0.763-0.784), respectively.
